# Report of the Obstetric Department of the Philadelphia Dispensary for the Year 1839

**Published:** 1840-01-25

**Authors:** Joseph Warrington

**Affiliations:** Accoucheur


*Report of the Obstetric department of the Philadelphia Dispensary for the year* 1839. By Joseph Warrington, M. D., Accoucheur.

Seventy-seven women have been under the care of the accoucheur, in this institution, during the year.

Seventy-nine children have been delivered, viz: 53 boys, and 26 girls; one woman having twin boys, and one twin girls.

In 47 cases, in which the presentations and positions of the fœtuses were carefully observed, they were as follows:—

Twenty-eight in the first position of vertex, 11 in 2d do., 4 in 4th do. 3 in 1st position of breech. 1 in 4th position of knees. The average duration of labour in 45 cases, was 11 hours, 26 minutes; the extremes being 30 minutes, and 31 hours.

The average time required for the spontaneous expulsion of the placenta in 51 cases, was 19 minutes, the extremes being 5, and 60 minutes. In 6 other cases, some manual aid was necessary to the delivery of this mass, after a retention for 30, 60, 90, 120 and 240 minutes respectively.

In each of these cases the delay of its delivery depended upon either atony of the uterus, or the manner in which the placenta presented at its orifice, and *not* to *adhesion* or *hour-glass contraction.*

The insinuation of the finger in 4 of the cases, and of the hand into the vagina and os uteri in two, was found sufficient for the completion of delivery.

In one case the fœtus presented originally in the 4th, but became spontaneously changed to the 2d position of the vertex.

In one case, flexion of the head upon the thorax did not take place until the hand was introduced, to assist the change.

The forceps were applied in four cases, viz.: in one which, 1 year and 15 days previously, had been delivered by the crotchet, in consequence of deficient amplitude of the pelvis, (child now delivered alive;) one in consequence of contraction of the antero-posterior diameter, with the additional obstacle of the jutting in of the left acetabulum, (child living, and case given in detail by Wm. H. Muller, one of the obstetric class;) in one, in consequence of an irreducible prolapsus of the cord, (child not quite dead when delivered, but could not be resuscitated,) and in a 4th case, in consequence of the defect of uterine action, and a small, but well formed pelvis—ergot having failed to effect delivery, (child living.)

Ergot was used in this one case only, during the whole year.

There were four cases of hæmorrhage,—two before labour had fairly begun; one during the early stage, and one several hours subsequent to delivery, in consequence of an attempt by the patient to rise from her bed.

In the first three cases it occurred apparently in consequence of the proximity of the placenta to the os uteri; one of these cases was accompanied with convulsions of a hysterical character.

Five children were still-born; one in consequence of an irreducible prolapsus of the cord; one from severe compression of the uterus—there being *no* liquor amnii within the membrane. The others appeared to have been dead some time previous to delivery, as the cuticle peeled off during their passage through the pelvis.

All the women recovered except one, who died of phthisis, eight days after delivery, which occurred three weeks before term,—the patient not being expected to live till that period. Delivery was effected chiefly by uterine efforts, the emaciated mother being scarcely conscious of the circumstance.

Two patients had metritis, coming on after natural and easy labours; and one other patient, after considerable manipulation and ultimate use of the forceps. These were all cured in a few days by bloodletting, general and local, moderate purgation, fomentations to abdomen, and mucilaginous injections into vagina.

There was one case of severe uterine and mammary engorgement, occurring on the third day after a natural labour; it was promptly cured by two doses of calomel and castor oil, and the frequent use of fomentations both to mammæ and abdomen.

One patient suffered much from ovaritis during the latter part of gestation. She was greatly relieved by cupping before delivery, and a free leeching subsequently.

In one case, attended by slight hæmorrhage after delivery, the placenta was found to be studded with numerous calcareous deposites upon its uterine surface. Several other placentæ were remarked to contain this species of formation, but no peculiar condition of the patient or child was noticed in connection with this circumstance.

Four or five of the children had ophthalmia; all of them recovered except one, which died in convulsions, eight days after its birth, the inflammation being intense, and no nurse could be obtained to attend upon the mother and apply the remedies proposed for the relief of the child.

Dr. Warrington holds the appointment of accoucheur to this ancient and extensive institution, with the view to establish a school of practical Midwifery. The cases are distributed amongst those members of his class who attend upon his course of practical instructions in Obstetrics, after having attended a full course of Anatomy and Midwifery in some respectable Medical School. Twenty-five gentlemen have participated in his course of instructions at the Dispensary, and attended upon the above list of cases during the past year.

The number of patients for 1839 has exceeded that for the previous year, by twenty-four.

